# Mutations within the tyrosine kinase domain of EGFR gene specifically occur in lung adenocarcinoma patients with a low exposure of tobacco smoking

**DOI:** 10.1038/sj.bjc.6603040

**Published:** 2006-03-21

**Authors:** K Sugio, H Uramoto, K Ono, T Oyama, T Hanagiri, M Sugaya, Y Ichiki, T So, S Nakata, M Morita, K Yasumoto

**Affiliations:** 1 Second Department of Surgery, University of Occupational and Environmental Health, 1-1 Iseigaoka, Yahatanishi-ku, Kitakyushu, 807-8555 Japan; 2 Department of Environmental Health, University of Occupational and Environmental Health, 1-1 Iseigaoka, Yahatanishi-ku, Kitakyushu, 807-8555 Japan

**Keywords:** EGFR, mutation, lung cancer, adenocarcinoma, smoking, screening, K-ras

## Abstract

Somatically acquired mutations in the epidermal growth factor receptor (EGFR) gene in lung cancer are associated with significant clinical responses to gefitinib, a tyrosine kinase inhibitor that targets EGFR. We screened the EGFR in 469 resected tumours of patients with lung cancer, which included 322 adenocarcinomas, 102 squamous cell carcinomas, 27 large cell carcinomas, 13 small cell carcinomas, and five other cell types. PCR with a specific condition was performed to identify any deletion in exon 19, while mutant-allele-specific amplification was performed to identify a mutation in codon 858 of exon 21. EGFR mutations were found in 136 cases (42.2%) with adenocarcinoma, in one case with large cell carcinoma, and in one case with pleomorphic carcinoma. An in-frame deletion in exon 19 was found in 62 cases while an L858R mutation was found in 77 cases. In the 322 cases with adenocarcinoma, these mutations were more frequently found in women than in men (*P*=0.0004), in well differentiated tumours than in poorly differentiated tumours (*P*=0.0014), and in patients who were never smokers than in patients who were current/former smokers (*P*<0.0001). The mutation was more frequently observed in patients who smoked ⩽20 pack-year, and in patients who quit at least 20 years before the date of diagnosis for lung cancer. The K-ras mutations were more frequently found in smokers than in never smokers, and in high-dose smokers than in low-dose smokers. In conclusion, the mutations within the tyrosine kinase domain of EGFR were found to specifically occur in lung adenocarcinoma patients with a low exposure of tobacco smoking.

Lung cancer is the leading causes of cancer-related death throughout the world, and also in Japanese from 1998. Although improvement of diagnostic technologies, more than 50% of patients present locally advanced or distant metastatic disease, which prognosis is still not satisfactory, because lung cancer is highly chemoresistant to most currently available chemotherapeutic agents. Therefore, the development of new modalities of treatment is important to improve the cure rate for lung cancer. The epidermal growth factor receptor (EGFR), which induces malignant tumour via three major mechanisms such as overexpression (Brabender *et al*, 2001; Hirsch *et al*, 2003), amplification (Shiraishi *et al*, 1989) and mutational activation (Downward *et al*, 1984), appears to be molecular target for therapeutic development. EGFR is a 170 kilodaltons (kDa) membrane-bound protein encoded by 28 exons on chromosome 7p12, and has a tyrosine kinase activity after binding of several specific ligands to the extracellular domain. These phosphorylated tyrosines lead to the activation of the downstream pathways of EGFR thus resulting in cell proliferation, differentiation, migration/motility, protection from apoptosis, or angiogenesis (Roskoski, 2004; Sordella *et al*, 2004; Baselga and Arteaga, 2005). EGFR-tyrosine kinase has become an attractive target for the treatment of non-small cell lung cancer (NSCLC), and agents targeting this receptor, including gefitinib, erlotinib, and cetuximab, are being investigated. Gefitinib is an orally active small molecule drug with evidence of an antitumour activity in NSCLC. In two clinical phase II trials, therapeutic response to the tyrosine kinase inhibitor (gefitinib) was predominantly observed in Japanese patients than European and US patients (Fukuoka *et al*, 2003; Kris *et al*, 2003). A clinical complete or partial response was especially observed most frequently in women, in nonsmoker, in patients with adenocarcinoma.

In 2004 three groups demonstrated the EGFR TK domain mutations in NSCLC and showed a striking correlation between the gefitinib sensitivity and TK domain mutations (Lynch *et al*, 2004; Paez *et al*, 2004; Pao *et al*, 2004). Subsequently, many reports demonstrated that the mutations of EGFR gene are detected in two specific regions such as an in-frame deletion in exon 19 and a missense mutation at the second nucleotide of codon 858 in exon 21 (Huang *et al*, 2004; Kosaka *et al*, 2004; Lynch *et al*, 2004; Paez *et al*, 2004; Pao *et al*, 2004; Shigematsu *et al*, 2005; Tokumo *et al*, 2005). *In vitro* studies expressing the alleles with these hot spot mutations suggest that mutant EGFRs selectively activate Akt and STAT signaling pathways, which promote cell survival, but have no effect on MAPK signaling, which induces proliferation (Sordella *et al*, 2004; Tracy *et al*, 2004). The ability to phosphorylate specific tyrosine residues in EGFR also appears to be inhibited by gefitinib or erlotinib at lower doses of drugs than that required for wild-type EGFR (Lynch *et al*, 2004; Paez *et al*, 2004; Pao *et al*, 2004). Theoretically, these mutations could all result in conformation changes that lead to increased activity as well as TKI sensitivity (Gazdar *et al*, 2004; Lynch *et al*, 2004; Paez *et al*, 2004). These EGFR mutations were significantly more frequent in East Asian patients than in Caucasian patients (Paez *et al*, 2004; Marchetti *et al*, 2005; Shigematsu *et al*, 2005). These two mutations were found in about 90% of all cases with EGFR mutations, therefore, we focused on two hot spots of mutation in EGFR TK domain.

In this study, we analysed the EGFR mutations in exons 19 and 21 by simple screening methods based on PCR in a large scale of Japanese patients with lung cancer, and investigated the clinical significance of these mutations.

## MATERIALS AND METHODS

### Patients

Tumour specimens were obtained from 469 consecutive patients with primary lung cancer and were stored according to protocols approved by the Institutional Review Board of the University of Occupational and Environmental Health and patients' written informed consent, from April 1996 to May 2005. The samples used in this study were obtained during surgical procedures from primary lung cancer patients with stage I–IV, according to the TNM classification revised in 1997 by the International Union Against Cancer (UICC). They included 322 adenocarcinomas, 102 squamous cell carcinomas, 27 large cell carcinomas, 13 small cell carcinomas, two pleomorphic carcinoma, one adenosquamous cell carcinoma, one carcinoid tumour, and one spindle carcinoma. They consisted of 302 men and 167 women ranging in age from 19 to 91 (average 66.2) years. One hundred seventy three had stage IA disease, 86 had stage IB, 11 had stage IIA, 54 had stage IIB, 73 had stage IIIA, 50 had stage IIIB, and 22 had stage IV disease. There were 138 never smokers and 331 ever smokers including 114 former and 217 current smokers (Table 1). In adenocarcinoma patients, there were 129 never smokers, 70 former smokers and 123 current smokers. Other clinicopathological features in patients with adenocarcinoma were shown in Table 2. The current smokers included patients who had stopped smoking less than 3 years previously, while former smokers meant that the duration since they quit smoking was more than 3 years.

### DNA extraction and sequencing analyses of the EGFR

Genomic DNA was extracted and purified from either fresh frozen tumours or tumours embedded in paraffin blocks. At first, in 20 samples, six exons of the TK domain (exons 18–23) were amplified using primers described in Lynch *et al* (2004) and uncloned PCR fragments were directly sequenced and analysed in both sense and antisense directions for the mutations using Applied Biosystems PRISM dye terminator cycle sequencing method with ABI PRISM 3100 (Applied Biosystems, Foster City, CA, USA)(Uramoto *et al*, 2006). The positive samples with mutations in exons 19 or 21 detected by sequencing were used as a positive control for screening methods described hereafter.

### Detection of in-frame deletion in exon 19 by simple screening method

**Figure 1:**
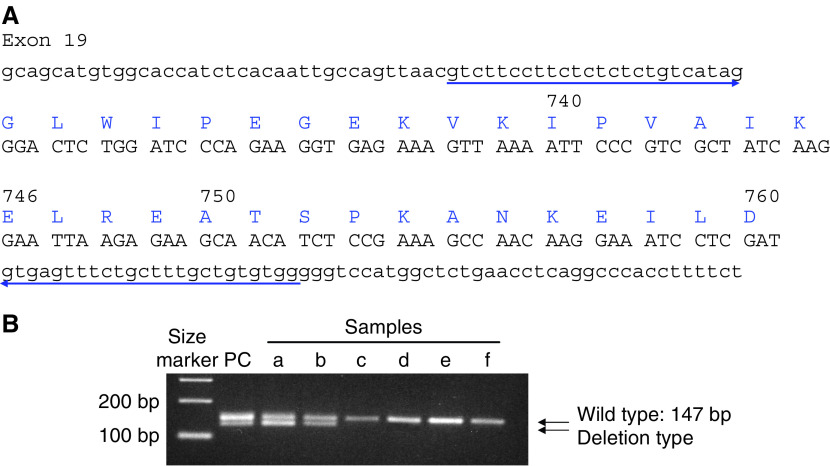
(**A**) PCR for detection of deletion in exon 19. The primers of PCR for detection of deletion in exon 19 were shown as arrow. (**B**) Agarose gel electrophoresis (4%). The PCR products were applied in 4% of agarose gel. The upper band shows wild-type allele as 147 bp, whereas shorter band was shown as a deletion allele in exon 19. PC: DNA as positive control which has 15 bp deletion (E746-A750del) confirmed by sequencing, a–b: positive samples for deletion, c–f: negative for deletion.

For detection of the in-frame deletion in exon 19, primers were constructed in order to make 147 bp product, when the allele was wild-type. The primer sequences are 5′-GTCTTCCTTCTCTCTCTGTCATAG-3′ as a sense and 5′-CCACACAGCAAAGCAGAAACTCAC-3′ as an antisense. PCR assay was carried out in 25-*μ*l reaction mixtures containing 1-*μ*l of genomic DNA using Taq DNA polymerase (TaKaRa Taq, TaKaRa, Shiga, Japan) for 35 cycles at 64°C for annealing, and the PCR products were run on electrophoresis in a 4% agarose gel containing 0.5 mg/ml ethidium bromide and visualised under UV (Figure 1A).

### Detection of point mutation in exon 21 by mutant-allele-specific amplification (MASA)

**Figure 2:**
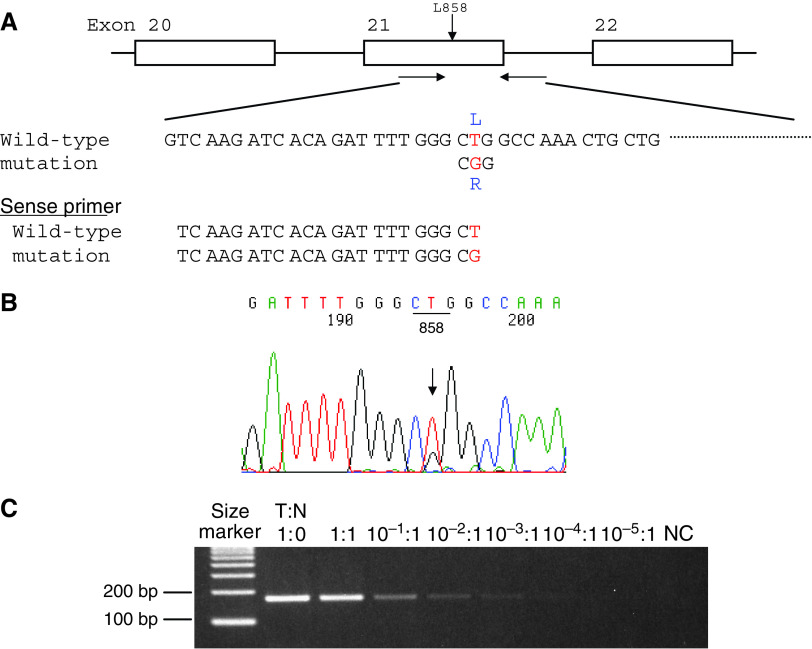
(**A**) Mutant-Allele Specific Amplification (MASA) for detection of a point mutation in exon 21. The primer sequences for detection of missense mutation (L858R) in exon 21 are shown. (**B**) Sequence analysis of lung cancer cell line (G603). Sequence analysis of lung cancer cell line (G603L) showed L858R mutation. The L858R mutation was detected in 4% agarose gel electrophoresis. (**C**) Sensitive assay for detection of L858R by MASA and agarose gel electrophoresis. DNAs derived from mixture of a cell line G603L (T) and a cell line (N) with wild-type EGFR were applied. A mutant allele was detected in the mixture of 10^−3^ diluted mutant DNA solution.

The 3′-ends of 22-bp oligonucleotides used as PCR primers corresponded to G for T of EGFR codon 858. That is, the sense-primer sequence for wild type was 5′-TCAAGATCACAGATTTTGGGCT, and that for L858R mutation was 5′-TCAAGATCACAGATTTTGGGCG. The antisense primer for both wild type and mutant type was 5′-CATCCTCCCCTGCATGTGTTAAAC (Figure 2). PCR assays were carried out for 38 cycles at 66°C for annealing. The PCR products were run on electrophoresis in a 2% agarose gel containing 0.5 mg/ml ethidium bromide and visualised under UV. To confirm the sensitivity of this method, exon 21 was amplified in the mixture of DNA solution of diluted mutation-positive DNA and wild-type DNA.

### Detection of K-ras mutation using PCR-based designed RFLP

For the detection of K-ras codon 12 mutations, we used our previously described designed RFLP method (Sugio *et al*, 1994; Sugio *et al*, 1997). Briefly, a sense-mismatched primer was used to introduce a new restriction site into the PCR product derived from wild-type allele. The newly introduced restriction sites were BstNI for screening for codon 12. Wild-type alleles were digested and they yielded a smaller product (77 bp) than mutant forms (97 bp), which were digestive-resistant. The sense-primer sequence was 5′-AAACTTGTGGTAGTTGGACCT, and the antisense primer was 5′-CTATTGTTGGATCATATTCG.

### Statistical analyses

We used the *χ*^2^ test and Fisher's exact tests to assess the relationship between EGFR gene mutations and each of the clinicopathological features. The Kaplan–Meier method was used to estimate the probability of survival, and survival differences were analysed by the log-rank test. All statistical tests were two sided, and *P*-values of less than 0.05 were considered statistically significant.

## RESULTS

### Exon 19 and 21 mutations in EGFR gene by screening method

PCR products in exon 19 revealed a 147 bp band when the allele was a wild type, and a shorter band when the allele was a deletion type, which were clearly separated in 4% agarose gel shown in Figure 1. In DNA samples which showed shorter band by this screening method, the exon 19 was amplified and directly sequenced. Next, the DNA showed an in-frame deletion, namely, a 15 base deletion from codon 746 to 750. We made 132, 135, and 138 bp PCR-products and analysed these products mixed with 147 bp product by agarose gel electrophoresis, as a result, a 12 bp difference was clearly detectable while 9 bp difference was suspicious. Therefore, this screening method was suitable to detect at least more than 12 bp deletion in exon 19.

To detect a point mutation of the second base of codon 858 in exon 21, MASA technique was performed as shown in Figure 2A. At first, exon 21 was amplified using a primer to detect a mutation of the second base of codon 858 for DNA samples with the mutation (L858R) or with only wild type, which was previously confirmed by sequencing. Agarose gel electrophoresis showed only the DNA sample with mutation to reveal a band, under stringent PCR conditions. Next, we used a DNA derived from cell line (G603L)(Sugaya *et al*, 2002) with an L858R mutation confirmed by sequencing (Figure 2B), as a positive control. To confirm the sensitivity of the MASA method, PCR was performed in the mixture of diluted mutation-positive DNA with wild-type DNA. As shown in Figure 2C, a mutant allele was detected in the mixture of 10^−3^ diluted mutant DNA solution, namely, an L858R mutation was detectable in one cancer cell with an L858R mutation of 10^3^ normal cells.

### EGFR mutations in tumour tissues of patients with lung cancer

In a total of 469 tumours of the patients who underwent a surgical resection, EGFR mutations were found in 136 cases (42.2%) with adenocarcinoma, in one case with large cell carcinoma, and in one case with pleomorphic carcinoma (Table 1). No other tumours including squamous cell carcinoma, small cell carcinoma, adenosquamous cell carcinoma, carcinoid tumour, and spindle cell carcinoma had these mutations. An in-frame deletion in exon 19 was found in 62 cases. All these samples showed a clearly separated band by agarose gel electrophoresis, which thus means a 12 or 15 bp deletion. An L858R mutation was found in 77 cases. One case had mutations in both exons 19 and 21.

### Relationship between EGFR mutations and clinicopathological features in adenocarcinoma

In the 322 cases with adenocarcinoma (Table 2), these EGFR mutations were more frequently found in female cases than in male cases (53.1 *vs* 33.5%, *P*=0.0004), in well-differentiated tumours than in moderately/poorly differentiated tumours (53.8 *vs* 36.4%, *P*=0.0014), and in patients who were never smokers than in patients who were smokers (57.4 *vs* 32.1%, *P*<0.0001). In smokers, the EGFR mutations were more frequently found in patients with former smokers than in patients with current smokers (44.3 *vs* 25.2%, *P*=0.0063). According to the pathological stage, no significant difference was found among the stages. An L858R mutation in exon 21 was more frequently found in female than male (*P*=0.0272), and in never smoker than smoker (*P*=0.0884). Sixty-two of the 76 female patients with mutations were never smokers, in which 40 patients (65%) had an L858R mutation.

**Figure 3:**
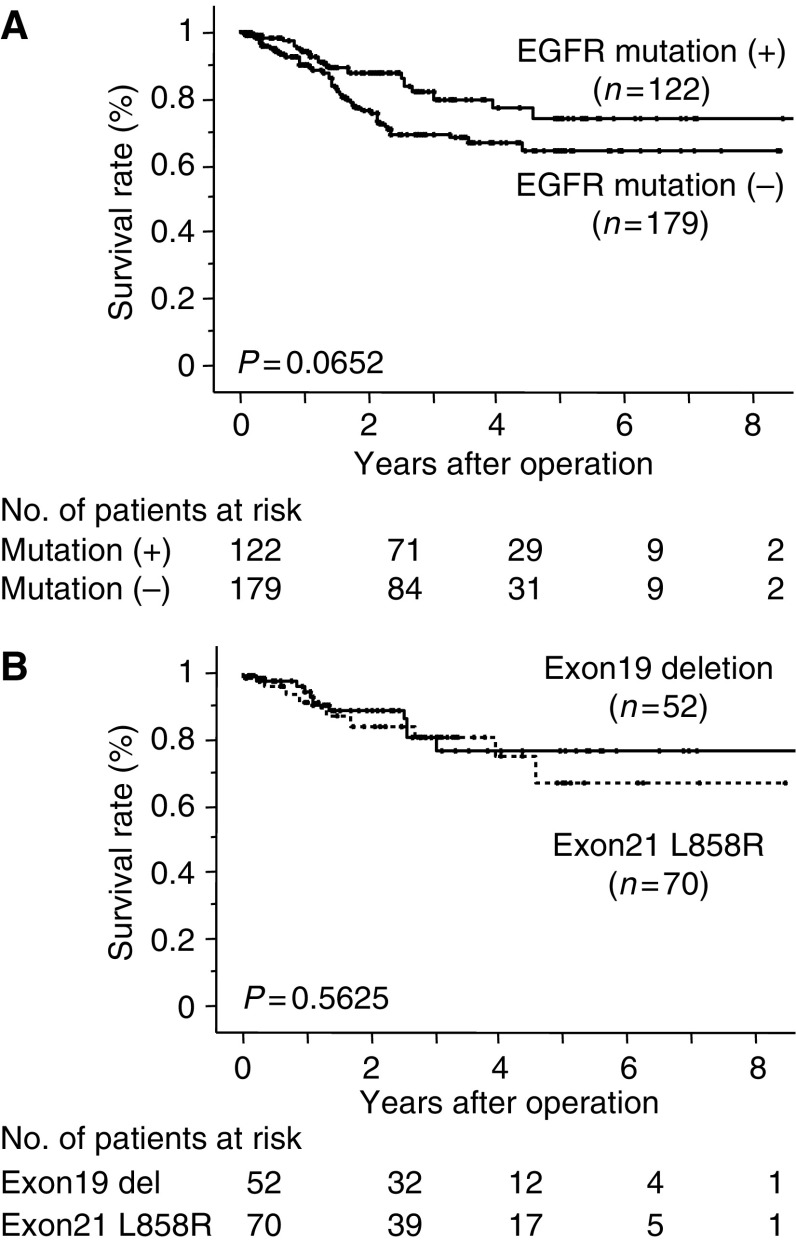
Kaplan–Meier survival curve for adenocarcinoma patients who did not receive gefitinib treatment. (**A**) The overall survival in relation to EGFR mutations in patients with adenocarcinoma. The five-year survival rate in the group with EGFR mutations and in the group without mutations was 73.6% and 64.1%, respectively, which did not show statistically significant difference (*P*=0.0652). (**B**) There was no statistically significant difference in the overall survival curves between patients with exon 19 deletion and exon 21 L858R (*P*=0.5625).

We examined the overall survival in relation to EGFR mutations in patients with adenocarcinoma who did not receive gefitinib treatment. The 5-year survival rate in the group with EGFR mutations and in the group without mutations was 73.6 and 64.1%, respectively, which did not show any statistically significant difference (*P*=0.0652) (Figure 3A). There was no statistically significant difference in the overall survival curves between the patients with exon 19 deletion and exon 21 L858R (*P*=0.5625) (Figure 3B).

### Relationship between EGFR mutations, K-ras mutation, and the smoking status in adenocarcinoma patients

In patients with adenocarcinoma, the mutation rate of EGFR in patients who were never smokers, in patients who had less than a 10 pack-year of smoking index, and in patients who had a 10–20 pack-year of smoking index was 57.4, 66.7, and 56.5%, respectively. However, EGFR mutations were less observed in patients who smoked more than 20 pack-year of smoking index, that is, the EGFR mutation rate in patients with 20–30 pack-year of smoking index, 30–60, 60–90, and more than 90 was 42.1, 25.0, 16.0, and 8.3%, respectively (Table 3). We analysed the mutations of K-ras codon 12 in patients with adenocarcinoma, and detected a mutation in 29 cases (9.0%), but none of them had the EGFR mutations (Table 2). Five of 129 patients who were never smokers had the K-ras mutation, while 24 of 193 patients who were current/former smokers had the mutation, which showed significant difference (*P*=0.0086) (Table 3). In patients who had less than a 60 pack-year of smoking index, 15 of 156 patients (9.6%) had the K-ras mutation, and in patients who had more than a 60 pack-year of smoking index, nine of 37 (24.3%) had this mutation, which showed significant difference (*P*=0.0148).

**Figure 4:**
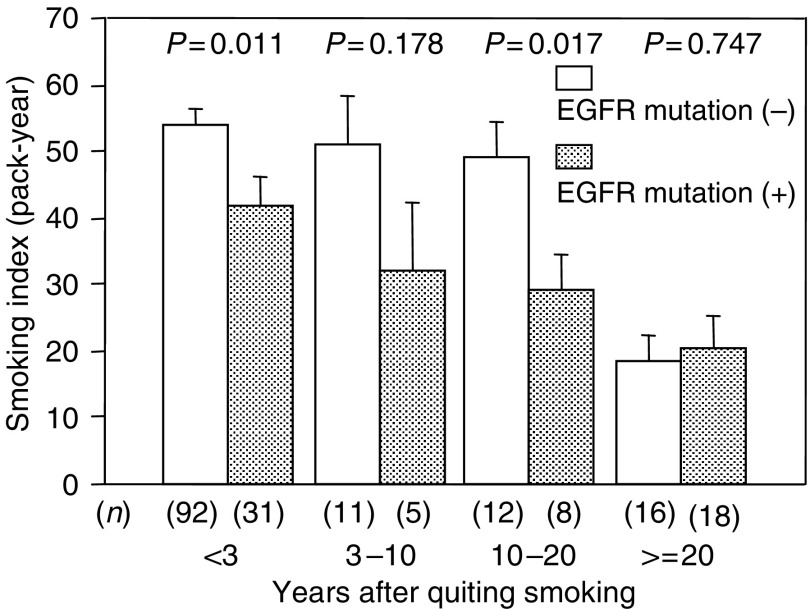
Smoking index in relation to the EGFR mutations stratified by the time duration of quitting smoking. The average smoking index in patients with EGFR mutations was lower than that in patients without EGFR mutations, and in the group less than 20 years after they had quit smoking. However, in the group that had quit smoking more than 20 years previously, no difference was observed in the average of smoking index between the patients with EGFR mutations and those without EGFR mutations.

We next examined the relationship between the EGFR mutations and time duration after the patients had quit smoking (Table 4). While the mutation rate of current smokers was 25.2%, the mutation rate of former smokers was 44.3%. In these former smokers, the mutation rate of the patients who stop smoking for 3–10 years, 10–20 years, and for more than 20 years was 31.3, 40.0, and 52.9%, respectively. The mutation rate of the patients who had stopped smoking for more than 20 years was almost same as that of the never smokers. On the other hand, the mutation rate of the K-ras codon 12 was not dependent on the time duration after the patients had quit smoking. Among current smokers, the smoking index was significantly higher in EGFR mutation-negative patients than in positive patients (*P*=0.011). The same results were also observed in former smokers who stopped smoking less than 20 years ago, however, no difference in the smoking index was found in patients who had stopped smoking more than 20 years previously (*P*=0.747) (Figure 4).

## DISCUSSION

In this study, we established a simple screening method to identify a deletion of exon 19 and a point mutation of exon 21 of the EGFR gene, and we detected these mutations in 136 cases (42.2%) with adenocarcinoma and two cases with other cell types. As shown in Table 5, in a total of 445 cases with the EGFR mutations from the previous reports which examined East Asian patients, 203 cases had an in-frame deletion in exon 19 and 188 cases had an L858R mutation in exon 21, namely in 90.0% of cases with EGFR mutations were detected either in exon 19 as a deletion and in exon 21 as a point mutation of L858R (Table 5) (Huang *et al*, 2004; Kosaka *et al*, 2004; Shigematsu *et al*, 2005; Sonobe *et al*, 2005; Soung *et al*, 2005; Tokumo *et al*, 2005). The mutant rate of exons 19 and 21 in all sites of mutations was observed relatively higher in Japanese patients than Korean patients, although the reason for this is unknown. Theoretically, these mutations could all result in conformation changes that lead to an increased activity as well as TKI sensitivity (Gazdar *et al*, 2004; Lynch *et al*, 2004; Paez *et al*, 2004). In an *in vitro* study, these two types of EGFR mutants demonstrated an enhanced TK activity in response to EGF and increased sensitivity to inhibition by TKI, although the biological activity between tumours with L858R and those with deletions was different (Paez *et al*, 2004; Pao *et al*, 2004). Therefore, detecting these two hot spot mutations is very useful to select a specific population which is sensitive to gefitinib treatment. On the other hand, a missense mutation in exon 20, especially T790M, showed gefitinib resistance, as previously reported (Kobayashi *et al*, 2005; Pao *et al*, 2005b). The T790M mutation detected in gefitinib-resistant tumours, was not found in any untreated tumours from the same patients. In resected tumours before treatment, only two of 397 tumours showed the T790M mutation (Toyooka *et al*, 2005). This mutation most likely extremely rare, or it might be present in some tumours at a low frequency at the time of diagnosis.

We used a simple method using PCR and agarose gel electrophoresis for the detection of a deletion in exon 19, and this method can detect more than a 12 bp deletion. Although the minimum deletion-size of exon 19 has been reported to be 9 bp, in previous studies (Kosaka *et al*, 2004; Paez *et al*, 2004; Shigematsu *et al*, 2005), this frequency was extremely low. Therefore, our method is useful for the screening of in-frame deletions of exon 19. The mutant-allele-specific amplification (MASA) method is capable of detecting one tumour cell containing genetic changes in a tumour sample containing thousands of normal cells (Takeda *et al*, 1993; Hayashi *et al*, 1994), to detect a point mutation. This method is very useful when the mutation occurs at a specific site, such as ras gene mutations which occur at the second base in codon 12 (Sugio *et al*, 1992; Sugio *et al*, 1994). Somatic mutations are detectable by a sequence analysis when the tumour has at least more than 5% of cancer cells. In some of the resected tissue samples or biopsy specimens, the ratio of cancer cell is less than 5%, and therefore, sensitive methods to detect such mutations are necessary. Pan *et al* (2005) reported sensitive assays based on a length analysis of fluorescently labeled PCR products for the detection of two predominant types of EGFR mutations, and thus showed four cases in which no mutations were apparent by sequencing. In this study, an L858R mutation was detectable in one cancer cell of 10^3^ normal cells, which is more sensitive than the previously reported method (Pan *et al*, 2005). The L858R mutation in exon 21 occurs in about 20–25% in adenocarcinoma of East Asian patients, therefore, this MASA method used in this study is useful for detecting cancer cells with a mutation in sputum, pleural effusion, or biopsy samples, when only a few cancer cells exist among a vast number of normal cells. This method might also be useful for the early detection of an acquired second point mutation at position 790 during gefitinib treatment (Kobayashi *et al*, 2005; Pao *et al*, 2005c), using appropriate primers. In addition, this sensitive method is simple and time saving for a routine pretherapeutic screening. Although the sequence analysis of EGFR might be necessary for clinical trials using TKI such as gefitinib or erlotinib, these simple methods established in this study for detection of exon 19 deletion and exon 21 L858R mutation are very useful for screening.

Previous studies have demonstrated EGFR mutations to be associated with adenocarcinoma, well differentiation, female gender, and never smokers (Kosaka *et al*, 2004; Lynch *et al*, 2004; Paez *et al*, 2004; Pao *et al*, 2004; Tokumo *et al*, 2005). In the present study, we identified EGFR mutations in 136 of 322 adenocarcinomas (42.2%) from Japanese patients, and confirmed these previous observations. However, we should keep in mind that the EGFR mutations are observed also in 30% of males and in 25% of current smokers. Adenocarcinomas in East Asian patients demonstrated a higher occurrence of EGFR mutations than in other ethnicities (Table 5) (Pao and Miller, 2005a; Shigematsu *et al*, 2005). In addition, the in-frame deletions in exon 19 were significantly more frequent in males while an L858R mutation in exon 21 was more frequently found in females, which is consistent with previous reports (Tokumo *et al*, 2005). We also demonstrated an inverse correlation between the EGFR mutations and exposure of tobacco smoking, namely, the smoking dose was closely related to the rate of EGFR mutations in adenocarcinoma, and we first showed a correlation between the time duration after the patients had quit smoking and EGFR mutations. The mutation rate of the patients who had stopped smoking more than 20 years previously was almost same as that of the never smokers, and no difference in the smoking index was observed between the patients with and without EGFR mutations. The Smoking effect is therefore very slight for oncogenesis via EGFR mutations of lung adenocarcinoma for the population who had stopped smoking for more than 20 years previously, whereas K-ras mutations were more frequently found in smokers as reported previously (Sugio *et al*, 1992; Ahrendt *et al*, 2001). As previously reported (Kosaka *et al*, 2004), both mutations between the EGFR and K-ras were also mutually exclusive in this study. Based on the smoking history of the patients, the K-ras mutations were more frequently found in smokers than in never smokers, and in high-dose smokers than in low-dose smokers. These findings demonstrated that the smoking dose was related to occurrence of the K-ras mutations.

Tobacco smoking is well-established high-risk factor for lung cancer, and therefore stopping smoking reduces the risk. In a large case–control study in the UK, the cumulative risks of lung cancer by 75 years of age are 15.9% for men who continue to smoke cigarettes and 9.9, 6.0, 3.0, and 1.7% for those who stopped smoking around 60, 50, 40, and 30 years of age, respectively. As a result, stopping smoking before middle age can allow an individual to avoid more than 90% of the risk attributable to tobacco (Peto *et al*, 2000). Although a low expose of smoking reduces the risk for lung cancer, EGFR mutations are suggested to be related to the occurrence of lung adenocarcinoma in nonsmoker.

Clinical trials with gefitinib have demonstrated good responses, particularly in patients with adenocarcinoma, and most frequently in females, nonsmokers, and East Asian patients (Fukuoka *et al*, 2003; Kris *et al*, 2003). Subsequently, it was demonstrated that the tumours with EGFR mutations are highly sensitive to gefitinib (Lynch *et al*, 2004; Paez *et al*, 2004; Pao *et al*, 2004) and the patients with EGFR mutations survived for a longer period after gefitinib treatment (Han *et al*, 2005; Mitsudomi *et al*, 2005). We also sequenced exons 18–23 of the EGFR gene of tumours in 20 patients with NSCLC who had been treated with gefitinib, and nine tumours had the mutations. Seven of nine cases with mutated types showed high sensitivity to gefitinib, and the patients with EGFR mutations had a more favourable prognosis than those with wild type after gefitinib treatment (*P*=0.033) (Uramoto *et al*, 2006). In previous clinical reports, gefitinib was effective in a few patients without EGFR mutations, thus indicating that not only mutations in TK domain but other mechanisms such as amplification, aberrant signaling may activate AKT and sensitise tumour cells to EGFR inhibitors (Amann *et al*, 2005; Takano *et al*, 2005). Although EGFR mutations itself is not a predictor for a better survival in adenocarcinoma patients as shown in both the present study and a previous study (Kosaka *et al*, 2004), EGFR mutations were good predictor of the clinical benefit with patients with gefitinib treatment in such patients.

In conclusion, our established methods are thus considered to be very useful for identifying a deletion of exon 19 and a point mutation (L858R) of exon 21 of the EGFR gene as a screening. The mutations within the tyrosine kinase domain of EGFR associated with gefitinib sensitivity were thus found to specifically occur in lung adenocarcinoma patients with a low exposure of tobacco smoking.

## Figures and Tables

**Table 1:** Clinicopathological features in relation to EGFR mutations in patients with lung cancer

**Variables**	**Category**	***n***	**EGFR mutation (%)**		**Exon 19 deletion**	**Exon 21 L858R**
Gender	Male	302	62^a^	20.5%	34	29
	Female	167	76	45.5%	28	48
						
Histology	Adenocarcinoma	322	136^a^	42.2%	62	75
	Squamous cell ca.	102	0	0.0%		
	Large cell ca.	27	1	3.7%		1
	Small cell ca.	13	0	0.0%		
	Pleomorphic ca.	2	1	50.0%		1
	Adenosquamous ca.	1	0	0.0%		
	Carcinoid tumour	1	0	0.0%		
	Spindle cell ca.	1	0	0.0%		
						
Smoking	Never	138	74^a^	53.6%	29	46
	Former^b^	114	31	27.2%	18	13
	Current	217	33	15.2%	15	18
						
	Total	469	138^a^	29.4%	62	77

a One case had mutations in both exons 19 and 21.

b Former smokers are defined persons who stopped smoking more than 3 years previously.

**Table 2:**
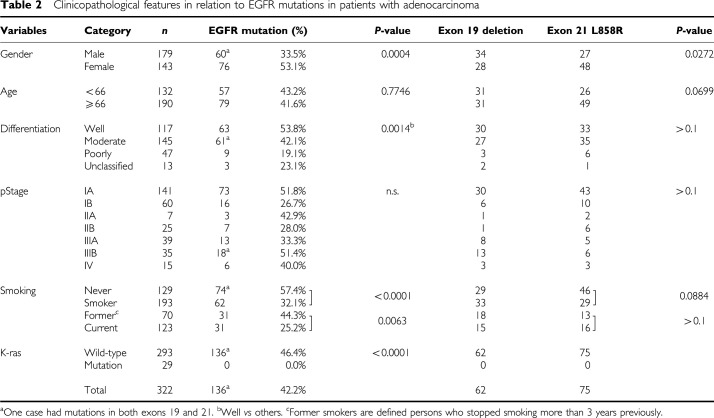
Clinicopathological features in relation to EGFR mutations in patients with adenocarcinoma

**Table 3:** EGFR and K-ras mutations in relation to the smoking index in patients with adenocarcinoma

**Smoking index (pack-year)**	***n***	**EGFR mutation (%)**	**K-ras mutation (%)**
0	129	74 (57.4)	5 (3.9)
⩽10	18	12 (66.7)	3 (16.7)
>10 ⩽20	23	13 (56.5)	2 (8.7)
>20 ⩽30	19	8 (42.1)	1 (5.3)
>30 ⩽60	96	24 (25.0)	9 (9.4)
>60 ⩽90	25	4 (16.0)	5 (20.0)
>90	12	1 (8.3)	4 (33.3)
			
Total	322	136 (42.2)	29 (9.0)

**Table 4:** EGFR and K-ras mutations in adenocarcinoma patients who were current and former smokers

**Years after stopping smoking**	***n***	**EGFR mutation (%)**	**K-ras mutation (%)**
<3^a^	123	31 (25.2)	13 (10.6)
⩾3<10	16	5 (31.3)	5 (31.2)
⩾10<20	20	8 (40.0)	2 (10.0)
⩾20	34	18 (52.9)	4 (11.8)
			
Total	193	62 (32.1)	24 (12.4)

a Current smokers are defined persons who stopped smoking less than 3 years previously.

**Table 5:** Incidence of EGFR mutations detected in East Asian patients with lung cancer without gefitinib treatment

**Author**	**Country or ethnicity**	**No. of tumours evaluated**	**No. of tumours with EGFR mutations (%)**	**No. of mutations of exon19 deletion or exon21 L858R (%)**	**No. of adenocarcinoma**	**No. of tumours with EGFR mutations (%)**
Kosaka *et al* (2004)	Japanese	277	111 (40.1)	101/111 (91.0)	224	110 (49.1)
Tokumo *et al* (2005)	Japanese	120	38 (31.7)	37/38 (97.4)	82	37 (45.1)
Sonobe *et al* (2005)	Japanese	154	60 (39.0)	56/60 (93.3)	108	60 (55.5)
Huang *et al* (2004)	Taiwanese	101	39 (38.6)	33/39 (84.6)	69	38 (55.1)
Soung *et al* (2005)	Korean	153	30 (19.6)	30/30 (100)	69	26 (37.7)
Shigematsu *et al* (2005)	East Asian	361	107 (29.6)	114/134 (85.1)^a^	214	102 (47.7)
Present study	Japanese	469	138 (29.4)	—	322	136 (42.2)
						
Total		1635	523 (32.0)	371/412 (90.0)	1088	509 (46.8)

a The total number of patients includes East Asian and other ethnicities.
